# Utilizing a Confronting Upside-Down Approach for IgG4-Related Posterior Mediastinal Fibrosis: A Report of a Rare Case

**DOI:** 10.7759/cureus.94867

**Published:** 2025-10-18

**Authors:** Kaiki Kawakita, Shuhei Iizuka, Tomonari Oki, Kambe Masato, Yoshiro Otsuki, Toru Nakamura

**Affiliations:** 1 Department of General Thoracic Surgery, Seirei Hamamatsu General Hospital, Hamamatsu, JPN; 2 Department of Pathology, Seirei Hamamatsu General Hospital, Hamamatsu, JPN

**Keywords:** igg4, immunoglobulin g4-related disease, mediastinal fibrosis, mediastinal neoplasms, thoracic surgery, video-assisted

## Abstract

IgG4-related disease (IgG4-RD) is a systemic fibroinflammatory condition mostly characterized by elevated serum IgG4 levels, organ involvement, and tissue fibrosis. Mediastinal fibrosis is a rare manifestation and is infrequently reported in clinical practice.

A 59-year-old man with progressive visual impairment underwent an evaluation, revealing an elevated serum IgG4 concentration of 1470 mg/dL. Orbital magnetic resonance imaging (MRI) showed an enlargement of multiple nerves, including the supraorbital and oculomotor nerves, and computed tomography (CT) identified a posterior mediastinal mass adjacent to the 6th-11th thoracic vertebral bodies without hilar or mediastinal lymphadenopathy. To obtain a definitive diagnosis, a biopsy was performed using the confronting upside-down video-assisted thoracoscopic surgery (VATS) approach. This technique, utilizing ports placed in higher intercostal spaces (ICS), provided optimal visualization and allowed safe and sufficient tissue sampling. Histopathological examination confirmed IgG4-positive plasma cell infiltration and fibrosis, meeting the diagnostic criteria for IgG4-related posterior mediastinal fibrosis. Five days post-surgery, oral prednisolone (PSL) therapy was initiated at 50 mg/day. This resulted in a significant improvement in visual acuity, with further recovery observed over three months as the PSL dose was tapered.

IgG4-related posterior mediastinal fibrosis is an uncommon condition, requiring biopsy for an accurate diagnosis. The confronting upside-down VATS technique ensures safe, precise tissue sampling with minimal complications, offering better visualization and access to challenging thoracic areas, making it a valuable approach for both biopsy and broader thoracic surgeries.

## Introduction

IgG4-related disease (IgG4-RD) is an immune-mediated condition involving multiple organs. It is characterized by elevated serum IgG4 concentration, infiltration of IgG4-positive plasma cells, and fibrosis, including storiform fibrosis, in the affected tissues. Commonly involved sites include the pancreas, biliary system, salivary glands, lacrimal glands, kidneys, and aorta. Mediastinal fibrosis is an uncommon manifestation of IgG4-RD, accounting for approximately 2.8% of reported cases [1-3].

The diagnosis is established as per the 2019 American College of Rheumatology/European League Against Rheumatism (ACR/EULAR) classification criteria for IgG4-RD [4]. These criteria require characteristic clinical, radiological, or pathological involvement of at least one typical organ, with exclusion of alternative conditions such as antineutrophil cytoplasmic antibody (ANCA)-associated vasculitis or collagen diseases with specific antibody positivity. The classification system comprises eight weighted domains encompassing pathological, serological, and organ-specific findings, and a cumulative score of ≥20 points is required for classification as IgG4-RD.

The challenging aspect of diagnosing IgG4-RD lies in its wide spectrum of clinical presentations, which often mimic other conditions. In many cases, tissue biopsy is necessary to obtain a definitive diagnosis. We herein report a case of IgG4-related posterior mediastinal fibrosis successfully biopsied using a confronting upside-down three-port video-assisted thoracoscopic surgery (VATS) approach.

## Case presentation

A 59-year-old man presented with progressive visual impairment. His past medical history included diabetes mellitus, managed with oral hypoglycemic agents, and fatty liver disease. Thirteen months prior, he was suspected of having a retinal artery occlusion and treated with 150 units of kallidinogenase daily, 1500 μg of Methycobal daily, and 1.25 mg of Nitorol spray daily for 30 days, all of which proved ineffective. Over the next 11 months, his visual acuity deteriorated, resulting in bilateral blurred vision. One month before presentation, his visual acuity was 1.8/6.0 in the right eye and reduced to light perception in the left eye.

**Figure 1:**
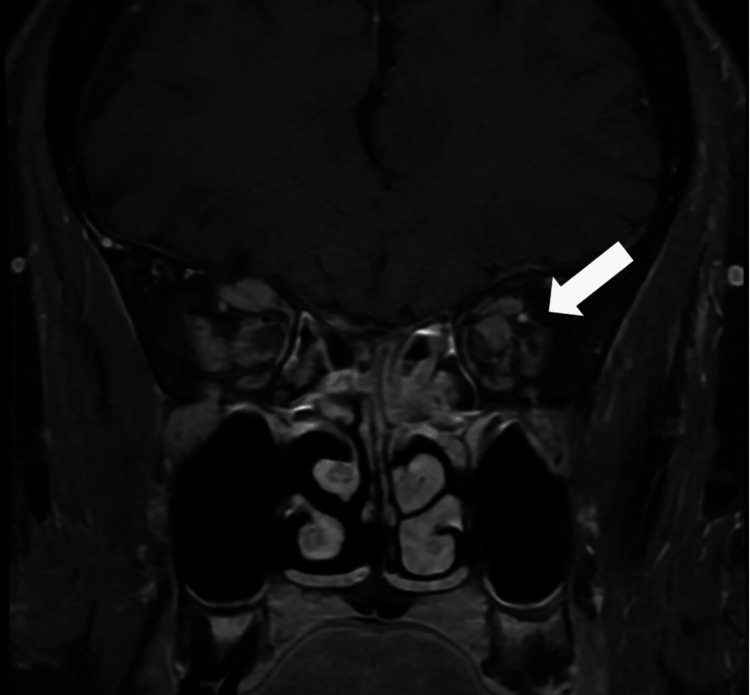
Orbital MRI Orbital MRI showing enlargement of both orbital nerves, with the superior branch of the oculomotor nerve compressing the left orbit nerve (arrow). MRI: magnetic resonance imaging

**Figure 2:**
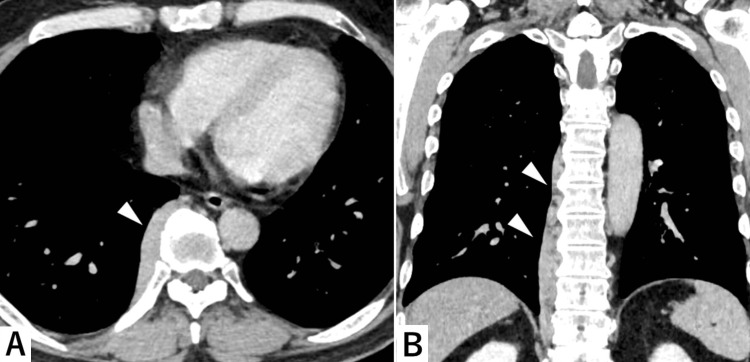
Chest CT scan CT scan (A, coronal view; B, axial view) showing a posterior mediastinal mass adjacent to the 6th-11th thoracic vertebral bodies (arrowheads). CT: computed tomography

An ocular fundus examination and optical coherence tomography ruled out retinal artery occlusion and diabetic retinopathy. Laboratory tests revealed an elevated serum IgG concentration of 1470 mg/dL. Orbital magnetic resonance imaging (MRI) revealed enlargement of multiple nerves, including the bilateral suborbital and supraorbital nerves, left nasal ciliary nerve, and superior branch of the oculomotor nerve, suggesting IgG4-related ophthalmic disease or mucosa-associated lymphoid tissue (MALT) lymphoma (Figure 1). Screening computed tomography (CT) revealed a posterior mediastinal mass adjacent to the 6th-11th thoracic vertebral bodies without hilar or mediastinal lymphadenopathy (Figure 2). No retroperitoneal or abdominal involvement was noted.

**Figure 3:**
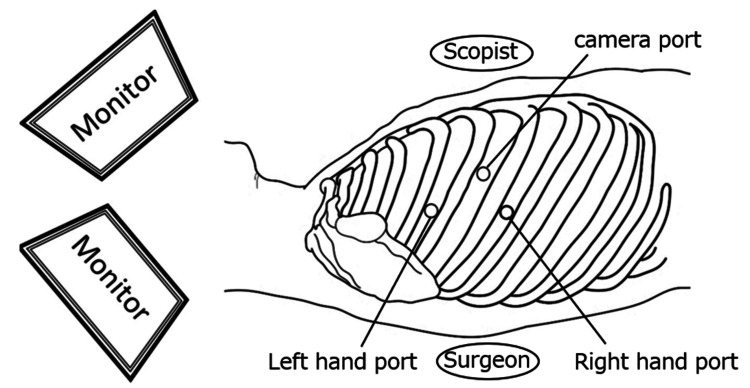
Port settings Port insertion sites and monitor settings for establishing the confronting upside-down VATS. A surgeon's left-hand port was placed in the 4th ICS along the posterior axillary line, a right-hand port in the 6th ICS along the posterior axillary line, and a camera port in the 5th ICS at the mid-axillary line. VATS: video-assisted thoracoscopic surgery; ICS: intercostal space Credits: Figure created by the authors

A surgical biopsy for the mediastinal mass was planned via VATS. With the patient in the left lateral decubitus position, the surgeon and the scopist stood facing each other with the patient in between. The scopist operated a thoracoscope (Olympus Medical Systems, Japan), examining the images, which were inverted up and down compared to those of the surgeon. A 5-mm utility port (Flexible Cannula, Karl Storz, Germany) was placed in the 4th intercostal space (ICS) along the posterior axillary line, an 11-mm port (Flexible Cannula, Karl Storz, Germany) was inserted in the 6th ICS along the posterior axillary line, and a 7-mm port (Flexible Trocar, Aesculap, Germany) was placed in the 5th ICS at the mid-axillary line as a camera port, establishing a confronting upside-down VATS setting (Figure 3) [5].

**Figure 4:**
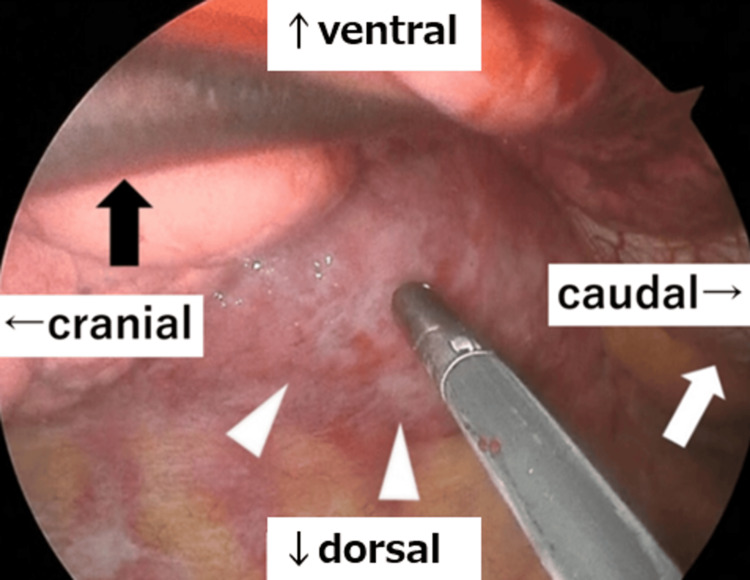
Operative view A tissue biopsy was obtained from the posterior mediastinal mass (arrowheads) using forceps in the surgeon's right hand, while the left hand (black arrow) retracted the lung ventrally. The white arrow indicates the diaphragm.

**Figure 5:**
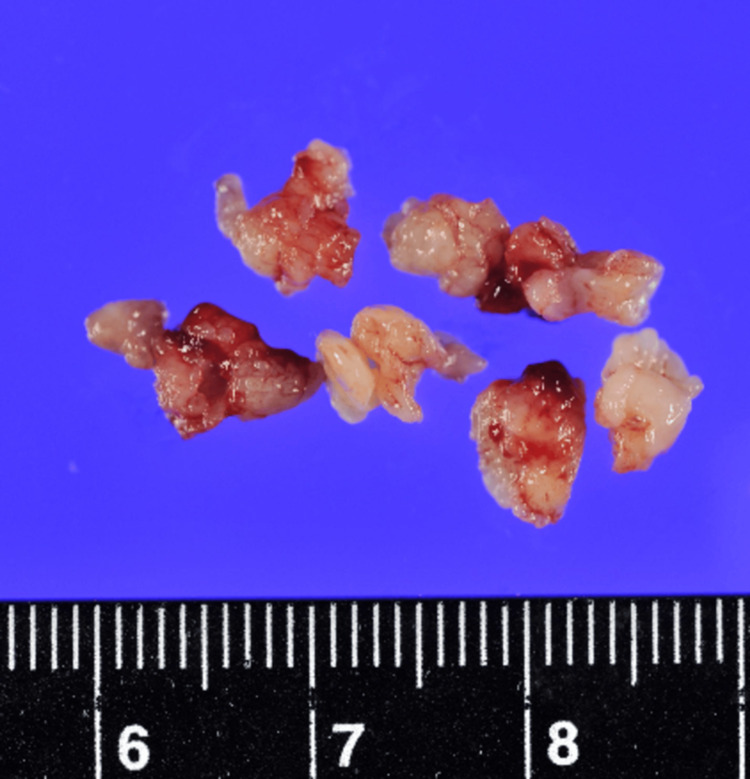
Gross findings of the surgical specimens Vascular-rich, elastic, hard nodules obtained from the posterior mediastinum.

Thoracoscopic findings revealed an irregular-margined, elastic, soft mass along the thoracic vertebral body. Using sponge forceps (Naruke Cotton Finger, Kenzmedico, Japan) in the left hand, the surgeon compressed the lung ventrally to maintain a clear surgical field while retrieving a sufficient specimen volume with biopsy forceps (Clickline Biopsy Punch, Karl Storz, Germany) in the right hand (Figure 4). The procedure concluded with complete hemostasis achieved within the same surgical field. The total operative time was 59 minutes, and the estimated blood loss was approximately 1 mL. Grossly, the lesion appeared as a vascular-rich, elastic, hard mass (Figure 5).

**Figure 6:**
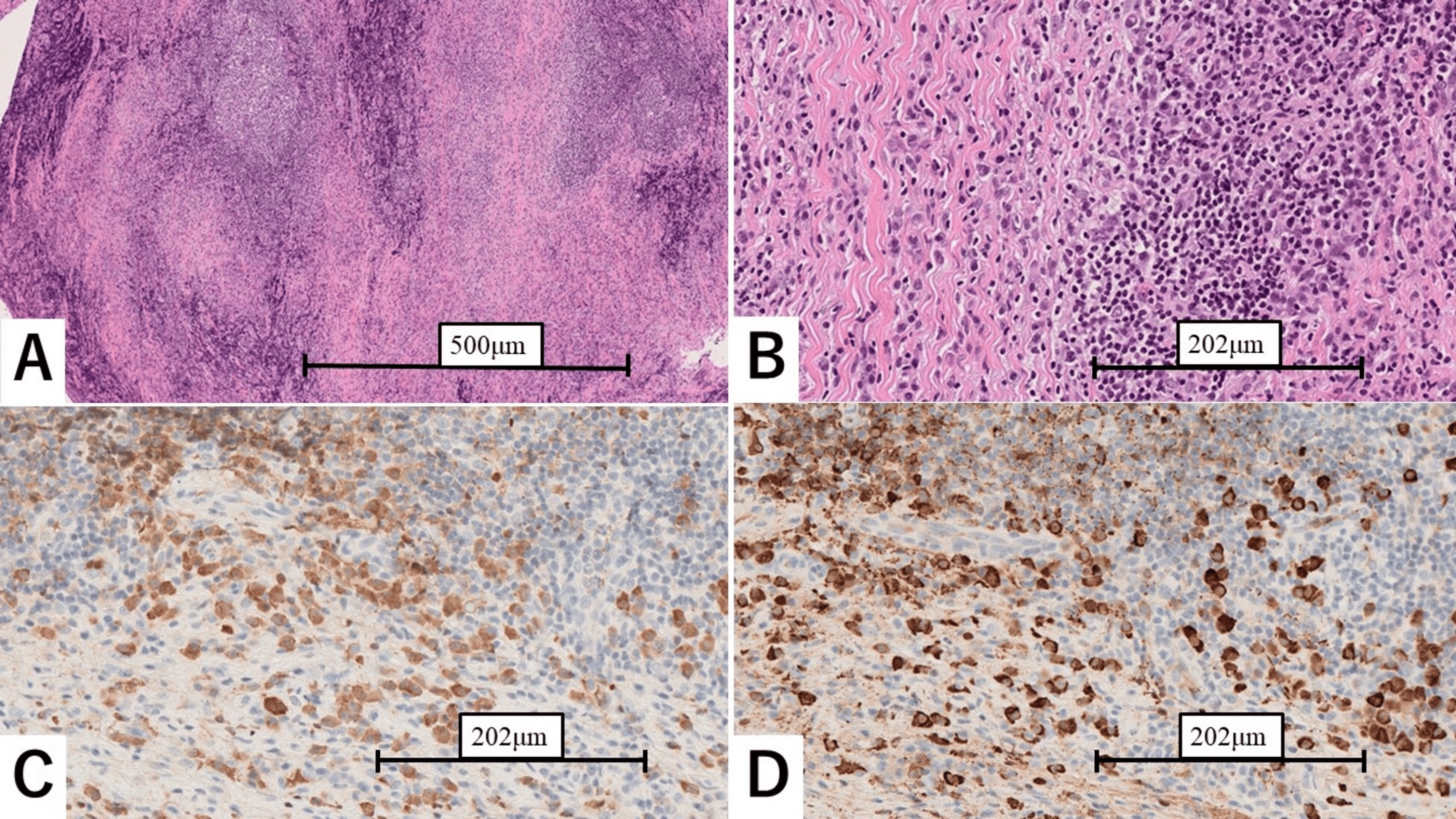
Histopathological findings (A) Low-power view (H&E staining, ×40) showing dense lymphoplasmacytic infiltration with partial formation of lymphofollicular structures. (B) Higher magnification (H&E staining, ×200) showing a mixture of lymphocytes and plasma cells with fibroblast proliferation. (C) Immunohistochemical staining for IgG (×200) highlighting numerous IgG-positive plasma cells. (D) Immunohistochemical staining for IgG4 (×200) revealing more than 125 IgG4-positive cells per HPF and an IgG4/IgG ratio of 98%. H&E: hematoxylin and eosin; HPF: high-power field

Pathological examination revealed fibrotic tissue infiltrated by numerous IgG4-positive plasma cells, with 125 IgG4-positive cells per high-power field and an IgG4/IgG ratio of 98% on immunostaining, fulfilling the ACR/EULAR criteria for a “definite” diagnosis (Figure 6). These findings were consistent with IgG4-related mediastinal fibrosis.

The patient was transferred to the neurology department on postoperative day two, and oral prednisolone (PSL) therapy was initiated on day five at a dose of 50 mg daily. The visual acuity in the left eye improved from light perception to finger counting at 2 m within six days of PSL initiation. Three months later, during tapering of the PSL dose to 10 mg per day, the best-corrected visual acuity improved to 7.2/6.0 in the right eye and 6.0/6.0 in the left eye.

## Discussion

IgG4-RD is a systemic fibroinflammatory condition marked by elevated IgG4 serum levels and multiple organ involvement. Although common sites include the pancreas, salivary glands, lacrimal glands, biliary tract, lungs, and retroperitoneum, mediastinal involvement is uncommon [6-8]. Among mediastinal cases, most are located in the left anterior mediastinum along the aortic arch [9-11], whereas posterior involvement presenting with a mediastinum fibrosis pattern is extremely rare [12]. Causes of posterior mediastinal fibrosis other than an IgG4-RD include a wide range of etiologies such as tuberculosis, aspergillosis, cryptococcosis, Behçet's disease, rheumatic fever, trauma, prior radiation therapy, and lymphoma [13,14]. Except for IgG4-RD, elevated IgG4 and optic nerve involvement are also seen in MALT lymphoma, a common orbital proliferative disorder [15]. The difficult point is that IgG4-RD and lymphoma may even coexist [5,16], suggesting that posterior mediastinal fibromas require a sufficient tissue sample for accurate diagnosis. 

Although a successful percutaneous CT-guided biopsy has been reported [12], we opted for a thoracoscopic biopsy to prioritize both safety and accuracy, as the tumor appeared too thin for a CT-guided diagnosis.

A sufficient specimen volume is required for the definitive diagnosis of posterior mediastinal fibromas, which are rare and require histological differentiation from many other diseases. The VATS approach is preferable to percutaneous needle biopsy due to safety concerns. Posterior intercostal artery (PIA) injury is a critical risk associated with thoracentesis for posterior mediastinal lesions near the vertebral body [17]. The biopsy needle trajectory must pass through the narrow ICS close to the vertebral body, increasing the risk of bleeding due to PIA injury. There have also been reports of PIA injury caused by paravertebral block for VATS [18]. Previous studies have emphasized the need to maintain a sufficient distance from the spinous process during dorsal intercostal puncture [19]. These findings suggest that thoracoscopic biopsy is preferable to percutaneous needle biopsy for the safe collection of adequate specimens from posterior mediastinal fibroma arising along the vertebral body.

Thoracoscopic approaches can be classified into the conventional looking-up method, in which a camera is inserted at an acute angle from a lower ICS with the surgeon and assistant sharing the same monitor, or the confronting upside-down method, where the camera is inserted from a higher ICS and separate monitors are used [3]. We chose the confronting upside-down method rather than the conventional looking-up approach as the surgical technique, allowing the camera to access the lesion at the shortest possible distance. The surgeon’s left-hand forceps effectively provided a sufficient field of view by compressing the lung ventrally, while the right-hand forceps enabled the secure retrieval of an adequate specimen volume and confirmation of hemostasis. These procedures were completed as a solo surgery without the need for an assistant. While the confronting upside-down technique has been primarily reported in anatomic lung resections, its application to mediastinal lesions near the lower thoracic vertebrae and diaphragm has also been described [20]. The present case further suggests the potential for expanding the application of confronting upside-down VATS to mediastinal surgeries beyond anatomical lung resections.

## Conclusions

Accurate diagnosis of IgG4-RD fundamentally relies on the identification of hallmark histopathological features, including dense lymphoplasmacytic infiltrates, storiform fibrosis, and an elevated number of IgG4-positive plasma cells, as specified in the 2019 ACR/EULAR classification criteria. Lesions located in the posterior mediastinum along the vertebral bodies pose particular challenges for tissue acquisition, and a VATS approach may be preferred over percutaneous biopsy, as it ensures procedural safety and sufficient tissue yield. In selected cases, the confronting upside-down technique can provide a viable alternative for accessing these posteriorly situated lesions. Accurate histopathological evaluation of such specimens is essential not only for definitive diagnosis but also for guiding appropriate clinical management. Given the rarity and complexity of IgG4-RD, a multidisciplinary approach integrating pathological, radiological, and clinical assessments remains crucial for optimizing patient outcomes and advancing understanding of this heterogeneous disease.
